# Baby, children, and adult biscuits. Differences in nutritional quality and naturalness

**DOI:** 10.1002/fsn3.3711

**Published:** 2023-09-25

**Authors:** Michelle Klerks, Sergio Román, Luisma Sánchez‐Siles

**Affiliations:** ^1^ Research and Development, Hero Group Murcia Spain; ^2^ Institute for Infant Nutrition, Hero Group Lenzburg Switzerland; ^3^ Marketing Department, Facultad de Economía y Empresa University of Murcia Murcia Spain

**Keywords:** additives, baby food, biscuits, child marketing, food naturalness, nutritional quality, sugar

## Abstract

This study examined and compared the nutritional quality and degree of naturalness between baby biscuits (<3 years), children biscuits (>3 years), and adult biscuits. Mintel's Global New Products Database was searched for “Baby Biscuits & Rusks” and “Sweet Biscuits/Cookies” (re)launched between July 2019 and July 2022 in four European countries (Germany, the Netherlands, Spain, and the United Kingdom), which resulted in 1280 products to be analyzed. Nutritional quality was measured by means of nutrient values per 100 g, and baby biscuits were assessed for compliance with the World Health Organization's latest Nutrient and Promotion Profile Model (NPPM). Degree of naturalness was measured using the food naturalness index (FNI). Baby biscuits had the best nutritional quality and were the most natural as compared to children and adult biscuits, but their energy density and sugar content require further attention. Nutritional quality was comparably poor in children and adult biscuits, and children biscuits were the least natural of the three groups. The NPPM requirements of not adding any free sugar at all to baby biscuits may drive parents to purchase alternative sweeter biscuits originally formulated and meant for children and adults. Reasonable regulations are needed to support product (re)formulations and to improve the current market food offer for babies and children.

## Graphical abstract

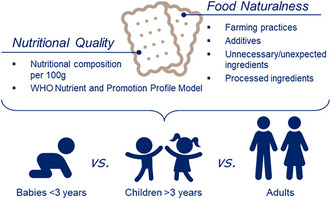

## 1 INTRODUCTION

A balanced and varied diet in the first years of life is crucial for the development of taste preferences and healthy eating habits that persist into later years (Langley‐Evans, 2015). Yet, there is increasing concern about the suitability of some commercially available products for infants, toddlers,^1^ and children (World Health Organization, 2022a). Biscuits are common snacks given to babies and children (Damen et al., 2019; Demonteil et al., 2019; Moore et al., 2019) and are frequently used in food marketing particularly aimed at children (Allemandi et al., 2020; Pombo‐Rodrigues et al., 2020; Román & Sánchez‐Siles, 2018). Criteria for the appropriate nutritional composition of baby biscuits (i.e., for infants and toddlers <3 years) exist, but they may differ per country or region. For instance, international global standards have been developed by Codex Alimentarius (2019), while in Europe requirements for baby food composition are laid down by European legislation (Commission Directive 2006/125/EC, 2006). When biscuits are targeted at older children (>3 years), this legislation does not apply anymore, and nutrient limits are no longer in place. Regardless of whether the nutritional composition is regulated or not, extensive research has shown that foods (including biscuits) targeted at babies (Elliott, 2011; Elliott & Conlon, 2014; Garcia et al., 2020; Grammatikaki et al., 2021; Hutchinson et al., 2021; Katiforis et al., 2021; McCann et al., 2022) as well as children (Beltrá et al., 2020; Elliott, 2019; Machado et al., 2019; Pombo‐Rodrigues et al., 2020; Richonnet et al., 2022; Santos et al., 2022) are high in nutrients of concern such as sugar, salt, and/or fat. In fact, both baby and child‐oriented foods are not always nutritionally different and in some cases, even worse than the adult equivalents (Beltrá et al., 2020; Cogswell et al., 2015; Elliott, 2011; Lapierre et al., 2016; Lythgoe et al., 2013; Machado et al., 2019).

A traditional basic biscuit usually consists of cereal flour, table sugar, fat/oil, and a raising agent (Arepally et al., 2020). However, according to NOVA's classification system, they are by definition ultra‐processed, due to the addition of sugar, fat/oil, and additives (Monteiro et al., 2019). Still, it is surprising that many baby biscuits across Europe have a “natural” or “free from artificial ingredients” claim (Grammatikaki et al., 2021). Since the term “natural” has not been regulated yet, the question remains as to how “natural” these products are according to the most objective approach to assess the degree of naturalness, namely the food naturalness index (FNI) (Sanchez‐Siles et al., 2019), and if there are differences compared to child‐oriented biscuits and adult biscuits.

Given these facts, baby and children's food manufacturers have been encouraged to reformulate their products and limit their marketing/advertising activities (Koo et al., 2018). Public health agencies such as World Health Organization (WHO) and Public Health England (PHE) have recently created nutritional guidelines for commercial baby foods (Public Health England, 2020; World Health Organization, 2022a). For instance, the WHO's latest Nutrient and Promotion Profile Model (NPPM) for infants and young children 6–36 months in the European Region and PHE's draft guidelines do not permit the addition of free sugar and sweetening agents in foods targeted at babies up to 36 months of age, and limits apply for salt and fat content. In fact, according to these organizations sweet snacks, like biscuits with added free sugars, should not be marketed as suitable for babies at all. Furthermore, the EU Pledge, a voluntary initiative by leading companies, establishes not to advertise food and beverage products to children under the age of 13 unless they meet specific nutrition criteria (EU Pledge, 2021).

As shown earlier, several studies have investigated the nutritional quality of commercial baby foods (<3 years) and children's foods (>3 years) and only few of them have compared baby or children's foods to adult foods. However, to the best of our knowledge, there are no studies comparing the nutritional quality and degree of naturalness of foods targeted at three different target populations (i.e., babies, children, and adults). Accordingly, the purpose of this study was to compare the nutritional quality and degree of naturalness and its components (farming practice, additives, unnecessary/unexpected ingredients, and processed ingredients) between recent launches of baby biscuits (<3 years), children biscuits (>3 years), and adult biscuits, available in four European countries. Additionally, added free sugar sources and the compliance of baby biscuits with the WHO's proposed NPPM were evaluated too.

## 2 MATERIALS AND METHODS

### 2.1 Data collection

Cross‐sectional data were collected using the Global New Products Database (GNPD) tool from Mintel Group Ltd. The database was searched for “Baby Biscuits & Rusks” and “Sweet Biscuits/Cookies” (re)launched between July 2019 and July 2022 in Germany, the Netherlands, Spain, and the United Kingdom, which are among the countries with the largest market volumes of biscuits, cookies, and crackers in Europe (Mintel, 2022). According to Mintel's category definitions, “Baby Biscuits & Rusks” includes all biscuits, rusks, and crackers positioned for babies and toddlers. “Sweet Biscuits/Cookies” includes cookies, sweet rice cakes, digestive biscuits, butter cookies, some chocolate covered biscuits/cookies, sandwich cookies, but also French macarons, sweet puff pastry twists, egg rolls, wafer rolls, and palmier cookies. For the scope of this research, “biscuits” had to be present in the product name to exclude products other than biscuits. Products with sweeteners falling under the “Sweet Biscuits/Cookies” category were also excluded, as these products made it impossible to have a fair comparison with baby biscuits, which by legislation, are not allowed to contain sweeteners (Commission Regulation (EC) No 1333/2008, 2008). The initial sample of baby biscuits was quite limited and was then expanded by the addition of biscuits of main baby food brands (which represent >70% of market share in snacks and biscuits) in order to have a larger and more balanced sample size in each country.

### 2.2 Sorting children and adult biscuits

Children biscuits were distinguished from adult biscuits by using the demographic filter “Children (5–12)” in the GNPD database. Mintel defines this demographic category as foods designed for the consumption by children, depending on presentation and format, such as child‐inspired graphics like cartoon characters, bright colors, pictures of children, and/or particular language. An additional manual sorting check was carried out to differentiate children biscuits from adult biscuits. Children biscuits were defined based on marketing techniques for packaging design to attract children, as in previous studies (Beltrá et al., 2020; Elliott, 2019; Lapierre et al., 2016; Machado et al., 2019; Mehta et al., 2012; Missbach et al., 2015; Savio et al., 2013). In particular, a child‐oriented biscuit had to meet at least two of the following criteria: (1) colorful packaging (e.g., multiple and/or bright colors), (2) child‐oriented text/brand or simply “child” or “kid” in the brand or product name, (3) promotional characters (e.g., cartoons, celebrities), (4) novel or unique packaging and/or food shapes, (5) references to play or education (e.g., games, puzzles), and (6) captions that exaggerated the attributes of the food (e.g., “dangerously cheesy”).

### 2.3 Data cleaning

**FIGURE 1:**
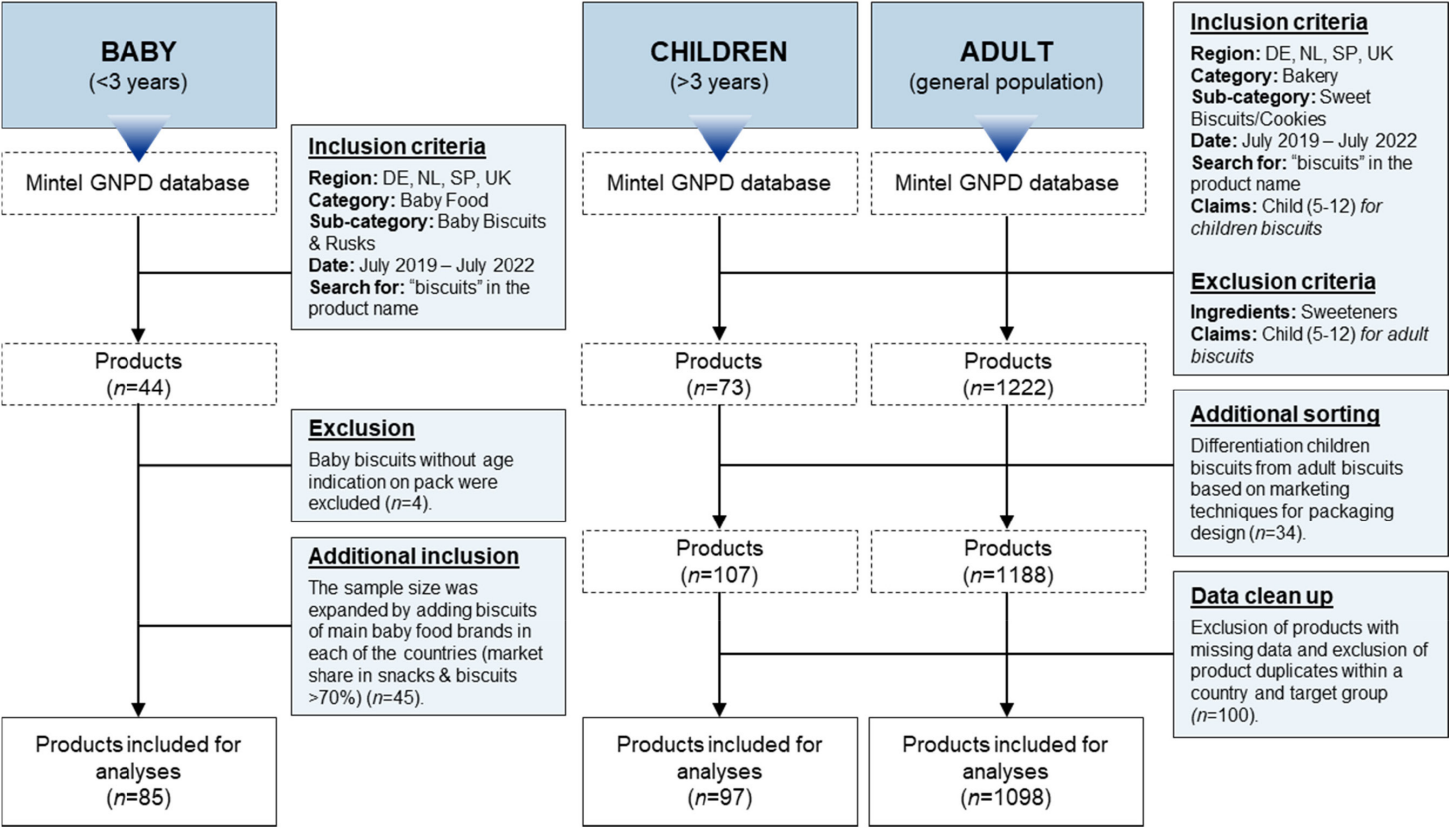
Data collection, sorting, and cleaning. Total sample biscuits *n* = 1280; baby biscuits (<3 years) *n* = 85, children biscuits (>3 years) *n* = 97, adult biscuits *n =* 1098.

Products that lacked the complete list of nutrient values, and product duplicates within a country and target group were identified and removed from the dataset. A product duplicate was considered when a product was released with a new packaging design, or when a recipe had been updated. In case of a product duplicate, the latest addition to the GNPD database was maintained. To avoid misclassification, baby biscuits without age indication were removed. Missing information regarding portion sizes was individually searched for on the products' websites. This procedure resulted in 1280 products to be included in analyses. See Figure 1 for an overview of data collection, sorting, and cleaning.

### 2.4 Nutritional quality

Nutritional quality was measured by means of nutrient values (energy, fat, saturated fat, carbohydrates, total sugars, fiber, protein, and salt) per 100 g. Since baby food is not eligible for the application of the Nutri‐Score (Sante Publique France, 2022), biscuits in this study were not evaluated with this nutritional labeling system. Instead, baby biscuits were checked for compliance with the proposed revised NPPM for commercially available complementary foods of the WHO European Region (World Health Organization, 2022a). In particular, baby biscuits were compared to the requirements set for the sub‐category “Dry or semi‐dry snacks and finger foods,” in terms of energy density (kcal/serve), sodium (mg/100 kcal), total sugar (%/energy), added free sugar or sweetener, total protein (g/100 kcal), total fat (g/100 kcal), and age on pack (months). Added free sugar or sweetener included (i) all mono‐ and disaccharides; (ii) all syrups, nectars, and honey; (iii) fruit juices or concentrated/powdered fruit juice, excluding lemon or lime juice; and (iv) non‐sugar sweeteners. The adherence to the criteria for added protein (i.e., “Biscuits, if made with high‐protein food, added protein must have ≥1.5 g/100 kcal”) could not be determined as quantities of possible high‐protein ingredients (e.g., milk or milk equivalent) were not quantified in the ingredient lists.

### 2.5 The food naturalness index (FNI)

Food naturalness was computed with the FNI, a validated methodology that is explained in more detail elsewhere (Sanchez‐Siles et al., 2019). The FNI considers the biscuit's farming practice, the number of additives, unnecessary/unexpected ingredients, and processed ingredients. All components could be derived from the information present on the product labels. To determine a product's farming practice, we evaluated whether the product carried an organic certification and/or age indication (<36 months) or not, in order to make a distinction between conventional, pesticide controlled (i.e., baby food grade), organic, and organic pesticide controlled (i.e., organic baby food grade) products. We have followed the definitions for each of the components as described in Sanchez‐Siles et al. (2019), but some additional adaptations and updates were made. In particular, mineral salts like calcium carbonate were counted as additives, unless it was clear from the label that it was added for fortification purposes. Sugar/sucrose, panela, demerara, and inverted sugar were not categorized as unnecessary/unexpected ingredients, as we considered sugar part of a traditional biscuit's recipe (Arepally et al., 2020). On the contrary, fruit juice concentrates not used for flavoring or coloring but only for sweetening purposes (e.g., grape juice concentrate, or apple juice concentrate when “apple” was not reflected in the product's name or packaging) were classified as unnecessary/unexpected ingredients. Concerning processed ingredients, a combination of different refined cereals (e.g., wheat flour, corn flour, rice flour) was considered as “refined cereals”. In the case of a combination of whole grain and refined cereals, the ingredients were not considered processed if the majority of cereals (≥50% of total cereals) were whole grain. Vanilla extract was excluded from the list of processed ingredients. Lastly, ingredients that were found multiple times in the ingredient list were only counted once in the corresponding components (e.g., “sugar” that is included in the formulation of the biscuit dough as well as in other compounds of the biscuit such as caramel pieces or chocolate), except for ingredients of which the exact type was unclear (e.g., “flavor” without designation of which flavor).

### 2.6 Data analysis

Statistical analyses were conducted in IBM SPSS Statistics version 27 (IBM Corp). Normality of the data was checked using the Shapiro–Wilk test. The normality of the data was rejected, and variables were expressed as median (Q1–Q3). Data of energy and nutrient content, and FNI and its related components, were tested for differences between the three target groups using the Kruskal–Wallis non‐parametric test for independent samples with multiple pairwise comparisons. The Bonferroni correction was applied and P‐values below 0.05 were considered significant. Microsoft Excel 365 was used to identify the different sources of added free sugars, additives, unnecessary/unexpected ingredients, and processed ingredients by using the IF function including multiple possible word options (e.g., “sugar” and “sucrose,” “lecithin,” and “E332”).

## 3 RESULTS

A total of 1280 biscuits from Germany, the Netherlands, Spain, and the United Kingdom were included in the analysis: 85 baby biscuits (6.6%), 97 children biscuits (7.6%), and 1098 adult biscuits (85.8%). Table 1 shows all biscuits analyzed per country and target group.

**TABLE 1:** Distribution of biscuits among countries and target groups.

Country	All biscuits	Baby biscuits	Children biscuits	Adult biscuits
	*n* (%)	*n* (%)	*n* (%)	*n* (%)
Germany	531 (41.5%)	27 (2.1%)	41 (3.2%)	463 (36.2%)
Netherlands	128 (10.0%)	15 (1.2%)	19 (1.5%)	94 (7.3%)
Spain	287 (22.4%)	17 (1.3%)	17 (1.3%)	253 (19.8%)
United Kingdom	334 (26.1%)	26 (2.0%)	20 (1.6%)	288 (22.5%)
All countries	1280 (100%)	85 (6.6%)	97 (7.6%)	1098 (85.8%)

*Note*: The number of biscuit launches in each country are consistent with the market volumes of biscuits, cookies, and crackers (Mintel, 2022).

### 3.1 Nutritional quality

**FIGURE 2:**
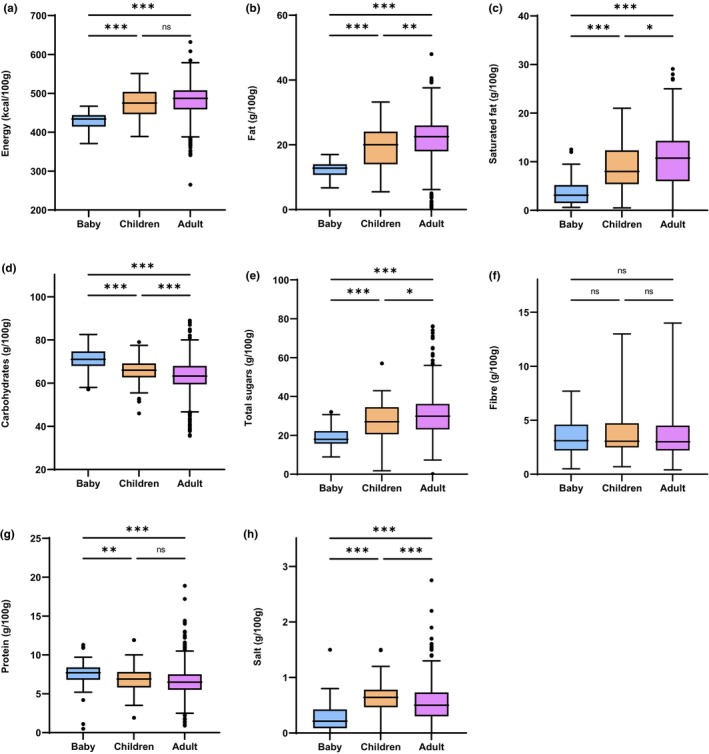
Boxplots of the nutritional composition of baby, children, and adult biscuits. (a) energy (kcal/100 g), (b) fat (g/100 g), (c) saturated fat (g/100 g), (d) carbohydrates (g/100 g), (e) total sugars (g/100 g), (f) fiber (g/100 g), (g) protein (g/100 g), and (h) salt (g/100 g). *p < .05, **p < .01, ***p < .001, ns = not significant.

A description of the nutritional composition of the biscuits is provided in Table 2 and Figure 2. Median sugar content was found to be significantly lowest in baby biscuits (18.0/100 g), followed by children and adult biscuits (27.0/100 g and 29.9/100 g, respectively). Saturated fat was also lower in baby biscuits (3.1/100 g), as compared to children (8.0/100 g) and adult (10.7/100 g) biscuits. Salt was lowest in baby biscuits (0.21/100 g), and highest in children biscuits (0.64/100 g).

**TABLE 2:** Nutritional composition per 100 g of biscuits and comparison between the three target populations.

	Baby biscuits (*n* = 85)	Children biscuits (*n* = 97)	Adult biscuits (*n* = 1098)	*p*‐value
Energy (kcal)	434.0 (414.5–444.2)^a^	475.0 (446.5–504.2)^b^	487.0 (459.0–508.0)^b^	<.001
Fat (g)	12.8 (10.7–14.0)^a^	20.0 (14.0–24.1)^b^	22.5 (18.0–26.0)^c^	<.001
Saturated fat (g)	3.1 (1.5–5.2)^a^	8.0 (5.4–12.3)^b^	10.7 (6.0–14.3)^c^	<.001
Carbohydrates (g)	71.0 (68.0–74.7)^c^	66.0 (62.7–69.2)^b^	63.3 (59.5–68.0)^a^	<.001
Total sugars (g)	18.0 (15.7–22.2)^a^	27.0 (20.6–34.5)^b^	29.9 (23.0–36.2)^c^	<.001
Fiber (g)	3.1 (2.2–4.6)	3.1 (2.5–4.7)	3.0 (2.2–4.5)	.636
Protein (g)	7.7 (6.8–8.4)^b^	6.9 (5.8–7.8)^a^	6.5 (5.5–7.5)^a^	<.001
Salt (g)	0.21 (0.09–0.43)^a^	0.64 (0.45–0.78)^c^	0.50 (0.30–0.73)^b^	<.001

*Note*: Values are expressed as median (25th–75th percentile). Kruskal–Wallis nonparametric test was used to compare the energy and nutrient values between the different target populations. Different superscript letters in the same row indicate significant differences.

Notably, 99.8% of all biscuits contained added free sugars. In baby biscuits, most common added free sugar source was sugar (47.1%), followed by fruit juice concentrates (42.4%). Almost all children and adult biscuits contained sugar (93.8% and 98.0%, respectively), often in combination with another sugar source such as glucose syrup, inverted sugar syrup, or glucose‐fructose syrup (Table 3).

**TABLE 3:** Top five added free sugar sources.

Baby biscuits (*n* = 85)	%	Children biscuits (*n* = 97)	%	Adult biscuits (*n* = 1098)	%
1. Sugar	47.1	1. Sugar	93.8	1. Sugar	98.0
2. Fruit juice concentrate^a^	42.4	2. Glucose syrup	24.7	2. Glucose syrup	26.2
3. Glucose syrup	5.9	3. Inverted sugar syrup	18.6	3. Glucose‐fructose syrup	19.5
4. Rice syrup	5.9	4. Glucose‐fructose syrup	12.4	4. Inverted sugar syrup	12.9
5. Honey	3.5	5. Dextrin	12.4	5. Lactose	9.1

^a^ Lemon juice concentrate was excluded.

As shown in Table 4, all baby biscuits (100%) complied with the criteria set for total fat, and those made with a high‐protein food (*n* = 32) all complied with the total protein limit. Nearly all baby biscuits (96.5%) complied with the required minimum age on the label of 6 months. Most baby biscuits adhered to the limit of 50 mg/100 kcal of sodium (87.1%), and only 30.5% were in line with the total sugar limit of ≤15%/energy. 36.4% of the baby biscuits that provided a suggested portion size (*n* = 33), contained the established energy content of ≤50 kcal/serve. None of the biscuits were formulated without added free sugar.

**TABLE 4:** Compliance of baby biscuits (<3 years) in line with the proposed NPPM for commercially available complementary foods of the WHO European Region (World Health Organization, 2022a).

Criteria for “dry or semi‐dry snacks and finger foods”^a^	All baby biscuits (*n* = 85)	Germany (*n* = 27)	Netherlands (*n* = 15)	Spain (*n* = 17)	United Kingdom (*n* = 26)
Energy density^b^ ≤50 kcal/serve	36.4% (12/33)	41.7% (5/12)	50.0% (2/4)	75.0% (3/4)	15.4% (2/13)
Sodium ≤50 mg/100 kcal	87.1% (74/85)	85.2% (23/27)	73.3% (11/15)	94.1% (16/17)	92.3% (24/26)
Total sugar ≤15%/energy	30.5% (26/85)	48.1% (13/27)	13.3% (2/15)	35.3% (6/17)	19.2% (5/26)
Added free sugar or sweetener None	0% (0/85)	0% (0/85)	0% (0/85)	0% (0/85)	0% (0/85)
Total protein^c^ ≤5.5 g/100 kcal	100% (32/32)	100% (11/11)	100% (7/7)	100% (4/4)	100% (10/10)
Total fat ≤4.5 g/100 kcal	100% (85/85)	100% (27/27)	100% (15/15)	100% (17/17)	100% (26/26)
Age on pack 6–36 months	96.5% (82/85)	100% (27/27)	100% (15/15)	94.1% (16/17)	92.3% (24/26)

^a^ This category includes any grain, starch, pulse/lentil, or root vegetable snacks such as cracker, bread, biscuit, pastry, cake, or pancake, etc. Includes rusks, crackers, and biscuits intended to be eaten dry or pulverized with liquid.

^b^ Portion size data is missing for *n* = 52.

^c^ Biscuits made with the addition of a high‐protein food (e.g., milk or milk‐equivalent) for *n* = 32. Only one biscuit was presented as such (e.g., in product name, or named/pictured on packet), but all 32 biscuits with high‐protein food were included in the analysis. [correction added on 5 October 2023, after the first online publication: Last column heading “United Kingdom” was added to Table 4]

### 3.2 Food naturalness

**FIGURE 3:**
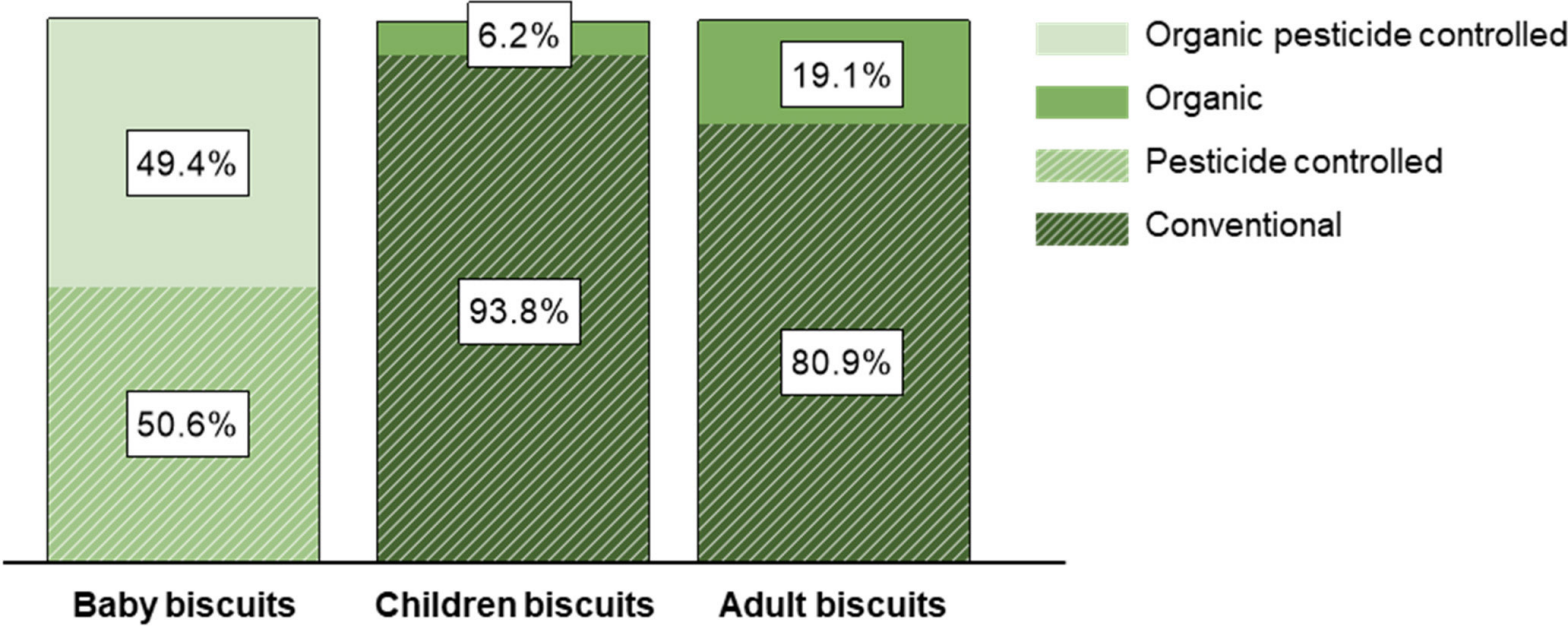
Distribution of farming practices (conventional, pesticide controlled, organic, organic pesticide controlled). Farming practices could be derived from on‐pack information (i.e., presence of age indication and organic certification logo) for all products.

Approximately half of baby biscuits were pesticide controlled, and half were organic pesticide controlled, while the majority of biscuits for children and adults were conventional (Figure 3).

Baby biscuits had the highest median FNI of 3.3 compared to 1.8 and 2.0 in children and adult biscuits, respectively (*p* < .001). Furthermore, baby biscuits had significantly the lowest scores in terms of additives, unnecessary/unexpected ingredients, and processed ingredients. On the contrary, children biscuits had significantly the highest scores in terms of additives and processed ingredients (Table 5).

**TABLE 5:** FNI and FNI components of biscuits and comparison between the three target populations.

	Baby biscuits (*n* = 85)	Children biscuits (*n* = 97)	Adult biscuits (*n* = 1098)	*p*‐value
FNI	3.3 (2.5–3.5)^c^	1.8 (1.4–2.0)^a^	2.0 (1.5–2.8)^b^	<.001
Number of additives	2.0 (1.0–3.0)^a^	4.0 (3.0–5.0)^c^	3.0 (2.0–5.0)^b^	<.001
Number of unnecessary/unexpected ingredients	1.0 (0.0–2.0)^a^	3.0 (1.0–4.0)^b^	2.0 (1.0–4.0)^b^	<.001
Number of processed ingredients	5.0 (4.0–6.0)^a^	9.0 (7.0–11.5)^c^	8.0 (5.0–11.0)^b^	<.001
Total number of ingredients	10.0 (8.0–13.0)^a^	17.0 (13.0–21.5)^b^	15.0 (11.0–20.0)^b^	<.001

*Note*: Values are expressed as median (25th‐75th percentile). Kruskal–Wallis nonparametric test was used to compare FNI and its components between the different target populations. Different superscript letters in the same row indicate significant differences. Total number of ingredients is not a component of the FNI, but generally used as an attribute for “clean label”. FNI scores: 1 = not natural at all, 2 = slightly natural, 3 = moderately natural, 4 = very natural, 5 = extremely natural.

As shown in Table 6, sodium carbonate and ammonium carbonate (raising agents), and lecithin (emulsifier) were the most common additives in all biscuits. Palm oil was the most frequently added unnecessary/unexpected ingredient in all biscuits. Children and adult biscuits shared a similar top 5 of unnecessary/unexpected ingredients, which included (other than palm oil) artificial flavor, wheat starch, glucose, and glucose‐fructose syrups. Fruit juice concentrates used for sweetening purposes were common unnecessary/unexpected ingredients in baby biscuits. Refined flour was the most frequently used processed ingredient in baby and children biscuits, whereas for adult biscuits it was sugar. Sunflower oil was a commonly used fat in baby biscuits, unlike palm oil that was more common in children and adult biscuits.

**TABLE 6:** Top 5 additives, unnecessary/unexpected ingredients, and processed ingredients.

Baby biscuits (*n* = 85)	%	Children biscuits (*n* = 79)	%	Adult biscuits (*n* = 1098)	%
Additives					
1. Sodium carbonate	74.1	1. Sodium carbonate	94.8	1. Sodium carbonate	77.2
2. Ammonium carbonate	38.8	2. Lecithin	81.4	2. Lecithin	58.2
3. Lecithin	24.7	3. Ammonium carbonate	59.8	3. Ammonium carbonate	51.1
4. Calcium carbonate	21.2	4. Citric acid	23.7	4. Citric acid	18.8
5. Potassium tartrate	16.5	5. Caramel color	7.2	5. Disodium diphosphate	8.2
Unnecessary/unexpected ingredients					
1. Palm oil	35.3	1. Palm oil	59.8	1. Palm oil	53.5
2. Fruit juice concentrate^a^	32.9	2. Artificial flavor	56.7	2. Artificial flavor	42.4
3. Wheat starch	23.5	3. Glucose syrup	24.7	3. Glucose syrup	26.2
4. Artificial flavor	15.3	4. Wheat starch	23.7	4. Glucose‐fructose syrup	19.5
5. Corn starch	10.6	5. Glucose‐fructose syrup	12.4	5. Wheat starch	19.1
Processed ingredients					
1. Refined flour	75.3	1. Refined flour	94.8	1. Sugar	98.0
2. Sunflower oil	57.6	2. Sugar	93.8	2. Refined flour	88.3
3. Sugar	47.1	3. Palm oil	59.8	3. Palm oil	53.5
4. Fruit juice concentrate	42.4	4. Artificial flavor	56.7	4. Artificial flavor	42.4
5. Skimmed milk powder	35.3	5. Skimmed milk powder	35.1	5. Natural flavor	30.5

^a^ Fruit juice concentrates not used for flavoring or coloring but only for sweetening purposes (e.g., grape juice concentrate, or apple juice concentrate when “apple” was not reflected in the product's name or packaging) were classified as unnecessary/unexpected ingredients. Lemon juice concentrates were excluded.

## 4 DISCUSSION

This is, to the best of our knowledge, the first study to compare the nutritional quality and degree of naturalness of baby, children, and adult biscuits in four European countries. Additionally, the most frequently added free sugar sources, additives, unnecessary/unexpected ingredients, and processed ingredients in all biscuits' formulations were investigated. The results showed that baby biscuits had the best nutritional quality and were the most natural as compared to children and adult biscuits. Furthermore, all baby biscuits complied with the WHO NPPM criteria in terms of fat, and the majority complied with the required age indication and sodium content. Yet, improvements need to be made in terms of energy density and sugar levels. Surprisingly, children biscuits, in comparison to adult ones, had higher levels of salt. In addition, they were the least natural of the three target groups, mainly due to the higher number of additives. Below we highlight our main findings along with their implications for health authorities, policy makers, and food manufacturers.

### 4.1 Nutritional quality evaluation of biscuits

Overall, the nutritional quality of baby biscuits in this study was very similar to recent findings from Grammatikaki et al. (2021) on 233 baby biscuits and rusks from 27 European countries. In contrast, all baby biscuits in our study contained added free sugar, while other studies (e.g., Grammatikaki et al., 2021; Hutchinson et al., 2021; Santos et al., 2022) found a percentage of products with no added free sugar. Also, some studies have described lower values of salt as compared to the present study (Elliott, 2011; Elliott & Conlon, 2014; Garcia et al., 2020; McCann et al., 2022). This might be explained by the small differences in the products tested; that is to say, the present study focused solely on baby biscuits, while the others examined “snacks,” “sweet dry snacks,” and “biscuits, rusks, and crackers” which not only included biscuits but also other snacks.

Notably, sugar content in our baby biscuit sample was much lower as compared to children and adult biscuits, however, there is room for improvement. For example, only <20% of baby biscuits in the Dutch and UK markets complied with the WHO sugar criteria of ≤15%/energy. Manufacturers should find ways to reduce the sugar levels as much as possible. However, sugar gives volume and texture to the product (Buttriss, 2013; Mamat & Hill, 2018), which facilitates melting and hence reduces the likelihood of choking. Hence, formulation of baby biscuits with sugar needs to be kept to a minimum, but should be allowed in the absence of other current viable alternatives. Otherwise, the latest recommendations from public health agencies (i.e., WHO, PHE) of not adding any free sugar at all to a baby biscuit formulation may drive parents to purchase alternative sweeter biscuits (as evidenced in our research) originally formulated and meant for children and adults. The WHO acknowledges this potential issue and stated that “Stringent requirements for added sugar in particular will render many snack products unmarketable (and not all can be reformulated). For example, rusk‐type biscuits […]” and “Gaps in the market may drive caregivers to purchase high sugar alternatives marketed for older children and adults” (World Health Organization, 2022b, p. 57). This would be particularly concerning as prior research has found that not only infants and toddlers consume family foods such as adult biscuits and cereals (Robinson et al., 2007; Roess et al., 2018), but also hyperpalatable foods, primarily through the exposure to adult foods (Kong et al., 2021).

Besides the quantity of sugar, the type of added free sugar and the presence of “hidden sugars” in baby food are being debated too. An example of “hidden sugars” is added fruit juice concentrates that are not clearly stated as sugars on pack, despite contributing to total free sugars. Fruit juice concentrates are being criticized and said to mislead parents who may not be aware of buying sugary options (Action On Sugar, 2021). In fact, consumers perceive a food to be healthier if the food is labeled with “fruit sugar” instead of “sugar” (Sütterlin & Siegrist, 2015). In our study, we found that fruit juice concentrates were the second most common added free sugar sources in baby biscuits which is in line with previous research (Cogswell et al., 2015; García et al., 2019; Hutchinson et al., 2021). Accordingly, confusion and misguidance among consumers would be reduced if manufacturers were more transparent about the fact that fruit juice concentrates are not nutritionally superior to table sugar, since both are classified as free sugars (EFSA Panel on Nutrition Novel Foods and Food Allergens (NDA) et al., 2022).

Our study also evaluated the recommended portion sizes on baby biscuits' packs in order to assess the compliance with the WHO NPPM criteria for energy density (kcal/serve). While doing so, two main findings need special attention. First, not many manufacturers provided a suggested portion size (only found on 39% of baby biscuits), which may result in portion sizes being decided by parents (and therefore not necessarily nutritionally adequate). Second, a large variability in portion sizes was observed for those biscuits that did carry a suggested portion size on pack. Therefore, we strongly believe that manufacturers should clearly provide a suggested portion size on pack in line with the WHO NPPM so as to prevent babies from overeating occasional food items such as biscuits. In this regard, given the median energy content of baby biscuits of 434 kcal/100 g in the current sample, and WHO's energy density requirement of 50 kcal/serve (World Health Organization, 2022a), an ideal portion size would be no more than 10 g.

While previous studies clearly found worse nutritional quality in child‐oriented foods as compared to adult foods (Beltrá et al., 2020; Lapierre et al., 2016; Lythgoe et al., 2013; Machado et al., 2019), the present study showed slightly but significantly worse (i.e., higher) values for carbohydrates and salt, but better (i.e., lower) values in terms of fat, saturated fat, and total sugar, in children biscuits as compared to adult biscuits. Although significant differences between children and adult biscuits were found for some nutrients, nutritional quality was found to be comparably poor in both groups. The low nutritional quality of children biscuits, in addition to the substantial gap in terms of sugar, saturated fat, and salt between baby and children biscuits are worrisome.

### 4.2 Naturalness assessment of biscuits

Our analysis showed that baby biscuits were the most natural. This result was expected, since according to EU legislation many additives and other substances are not allowed in commercial baby foods (Commission Directive 2006/125/EC, 2006; Commission Regulation (EC) No 1881/2006, 2006). Farming practices were one of the main drivers in this regard as baby biscuits were either pesticide controlled or organic pesticide controlled. This implies stricter limits for the amount of pesticide residues applied as compared to conventional children or adult biscuits (Commission Directive 2006/125/EC, 2006; DeMaria & Drogue, 2017). Furthermore, baby biscuits were more natural thanks to a cleaner label in general; in other words, they had significantly the lowest number of additives, unnecessary/unexpected ingredients, and processed ingredients. On the contrary, children biscuits were the least natural, which could mainly be attributed to the high number of additives. Indeed, the usage of multiple raising agents, emulsifiers, and colorants was highly prevalent in children biscuits. Also, they were characterized by combinations of several sugars, fats, and artificial flavors. Overall, this may result in highly palatable and sensory‐appealing biscuits with low levels of nutritional quality and naturalness which should be closely monitored by health authorities.

Taking in mind the basic formulation of a biscuit (Arepally et al., 2020), there are certain ways for manufacturers to improve their degree of naturalness such as the replacement of refined flours for whole grain flours, adding extra virgin olive oil or a cold‐pressed oil instead of palm oil, lowering the amount of sugars and additives to one, and/or avoiding the use of starches and artificial flavors (Sanchez‐Siles et al., 2019).

### 4.3 Strengths, limitations, and future research

The present study should be interpreted in view of its strengths and limitations. First, our analysis relied on a dataset of biscuits launched in the last 3 years in four European countries. Although such recent launches reflect the trends in new product developments and reformulations, they do not necessarily represent the best‐selling biscuits per se. We encountered that the sample size of recent baby biscuits launches was small and did not entirely mirror the current market, hence we enlarged the sample size by including biscuits from best‐selling brands, which accounted for >70% of the market share in each country. Second, our study is strengthened by the fact that an additional and manual sorting task was carried out to make a distinction between children and adult biscuits based on child‐oriented marketing techniques used for packaging design. This procedure allowed us to identify 34 children biscuits that were initially grouped as adult biscuits by Mintel. Third, this study is the first to objectively measure the degree of naturalness of baby, children, and adult biscuits. Our results further prove that the FNI is a valuable tool to evaluate the degree of naturalness in line with recent results from various product categories (Klerks et al., 2022; Michel et al., 2021; Sanchez‐Siles et al., 2022). Nevertheless, in line with the limitations described earlier (Klerks et al., 2022), some ingredients could not be found in the predefined list of unnecessary/unexpected ingredients as specified in Sanchez‐Siles et al. (2019). Thus, consistent adaptations and updates were made. Future research needs to further evaluate consumers' perception on unnecessary/unexpected ingredients in different food categories, so that the “inclusion” and “exclusion” criteria of these ingredients could be expanded. Finally, future studies could evaluate and compare the nutritional quality and degree of naturalness of other foods frequently given to babies and children such as breakfast cereals, savory snacks, and dairy products, which would provide valuable insights for product (re)formulations towards healthier and more natural foods.

## 5 CONCLUSION

In conclusion, although baby foods, in particular sweet snacks like biscuits, are highly being criticized by policy makers, findings from the current study evidence that they are a healthier and more natural alternative to both children and adult biscuits. Nonetheless, baby food manufacturers need to continue their efforts in improving the nutritional composition of their products, for example, by reducing sugar levels while maintaining desirable sensory characteristics (e.g., texture). Suggested portion sizes in line with the required energy density should be clearly stated on pack so that biscuits do not displace meals. This study has demonstrated a big gap between baby biscuits (<3 years) and biscuits marketed at older children (>3 years). Children biscuits were nutritionally poor and not naturally formulated. To protect children from an unhealthy food environment, and to enable children to develop healthy eating habits, there is an urge and opportunity for manufacturers of child‐oriented biscuits to narrow the market gap by drastically improving their offer toward nutritionally balanced and natural food products. Strict, but realistic, regulations need to be in place to support product (re)formulations and to improve the current market offer for babies and children.

## AUTHOR CONTRIBUTIONS

**Michelle Klerks:** Conceptualization (supporting); data curation (lead); formal analysis (lead); investigation (equal); methodology (equal); software (equal); visualization (lead); writing – original draft (lead); writing – review and editing (equal). **Sergio Román:** Conceptualization (equal); methodology (supporting); validation (equal); writing – review and editing (lead). **Luisma Sánchez‐Siles:** Conceptualization (lead); methodology (equal); resources (lead); supervision (lead); validation (lead); writing – review and editing (equal).

## FUNDING INFORMATION

The authors declare that no funding or grant was obtained for the study.

## CONFLICT OF INTEREST STATEMENT

Michelle Klerks and Luisma Sánchez‐Siles are members of the Research & Development Department of the Hero Group, a Swiss international food manufacturer. Sergio Román declares no conflict of interest.

## Data Availability

Additional information or data related to the study shall be made available on request.

## Footnotes

^1^
For the sake of brevity, in this paper, “infants” and “toddlers” are together referred to as “babies.”
